# The Epidemiology of COVID-19 and MS-Related Characteristics in a National Sample of People With MS in China

**DOI:** 10.3389/fneur.2021.682729

**Published:** 2021-05-28

**Authors:** Yao Zhang, Hexiang Yin, Yan Xu, Tao Xu, Bin Peng, Liying Cui, Shuyang Zhang

**Affiliations:** ^1^Center of Multiple Sclerosis and Related Disorders, Beijing, China; ^2^Department of Neurology, Peking Union Medical College, Peking Union Medical College Hospital, Chinese Academy of Medical Sciences, Beijing, China; ^3^Department of Epidemiology and Biostatistics, Institute of Basic Medical Sciences, School of Basic Medicine, Peking Union Medical College, Chinese Academy of Medical Sciences, Beijing, China; ^4^Neurosciences Center, Chinese Academy of Medical Sciences, Beijing, China; ^5^Department of Cardiology, Peking Union Medical College, Peking Union Medical College Hospital, Chinese Academy of Medical Sciences, Beijing, China; ^6^National Rare Diseases Registry System of China, Beijing, China

**Keywords:** multiple sclerosis, COVID-19, disease modifying therapies, teriflunomide, immunosuppressant, relapse

## Abstract

Few studies have focused on immune status and disease activity in MS patients during the coronavirus disease 2019 (COVID-19) pandemic. The aim of this study is to investigate immune status, COVID-19 infection, and attacks in MS patients during the pandemic. An online questionnaire about COVID-19 infection, MS attack, and MS treatment during the pandemic was administered to all 525 MS patients registered in our hospital database from January 1, 2011, to June 1, 2020. Only 384 responded, of which 361 patients could be included in the final analysis. During the pandemic, 42.1% of the 361 patients and 65.0% of the 234 patients on immunotherapies were exposed to teriflunomide. Compared to patients who didn't receive treatment, patients exposed to DMTs had significantly lower levels of neutrophils (*P* < 0.01) and immunoglobulin G (*P* < 0.01), and patients exposed to immunosuppressants had significantly lower levels of immunoglobulin G (*P* < 0.05). Over 80% of our patients followed effective protective measures and none of the 361 MS patients in our cohort contracted COVID-19. Patients whose treatment was disrupted had a significantly higher annualized relapse rate (ARR) during than before the pandemic (*P* < 0.01), while the ARR of patients with continuous treatment or without treatment remained unchanged. During the pandemic, the risk of MS attack due to treatment disruption possibly outweighs the risk of COVID-19 infection under preventive measures, and MS treatment maintenance might be necessary.

## Introduction

**Figure 1:**
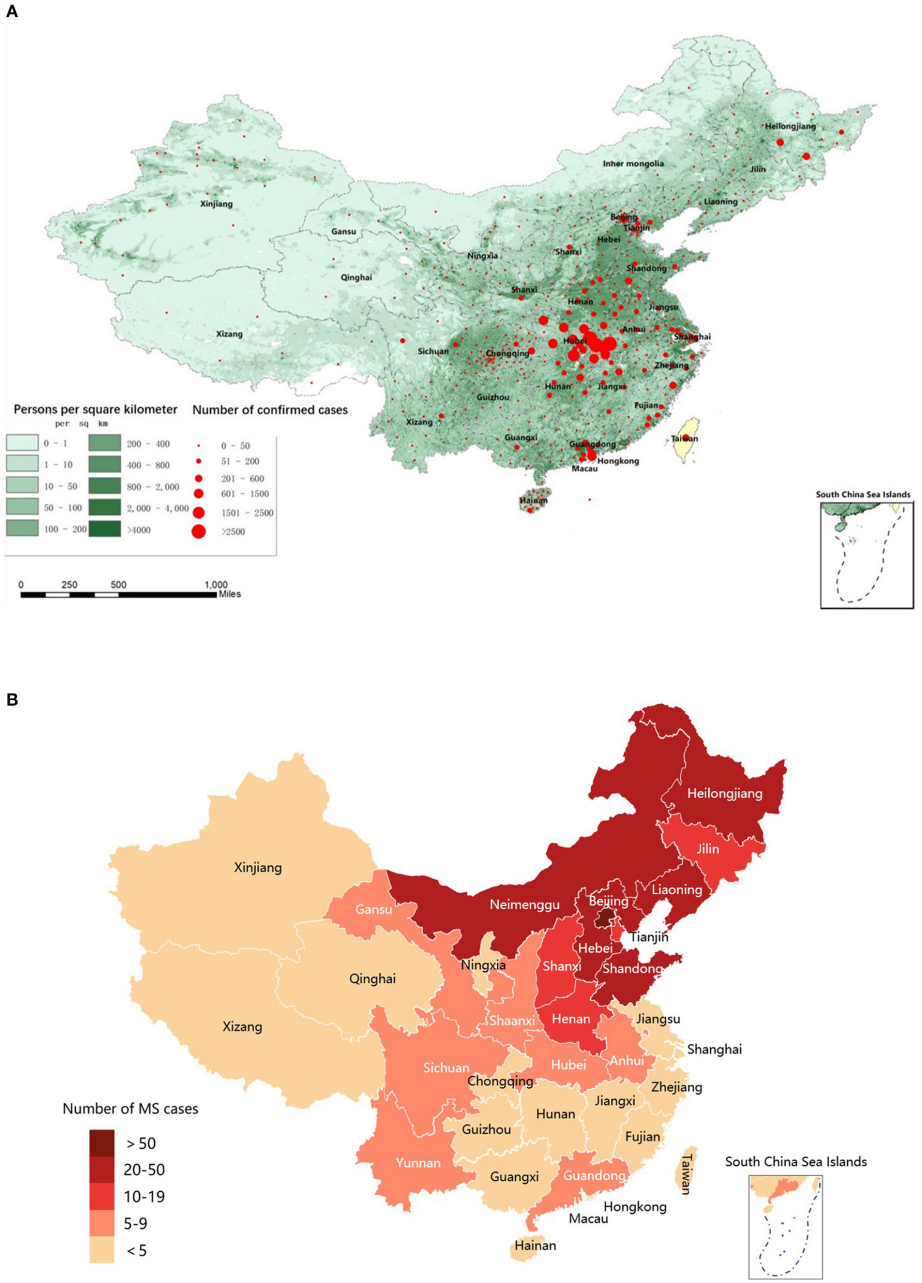
Distribution map of confirmed COVID-19 cases across China, population density in mainland China, and MS patients in the MS/NMO database. **(A)** Population density of China and distribution map of confirmed COVID-19 cases across China during the pandemic (From December 15, 2019, to June 1, 2020). **(B)** Distribution map of MS patients in the MSNMOBase across China. COVID-19, coronavirus disease 2019; MS, Multiple Sclerosis.

Coronavirus disease 2019 (COVID-19), caused by the severe acute respiratory syndrome coronavirus type-2 (SARS-CoV-2), has quickly developed into a global pandemic.^1^ As of June 1, 2020, a total of 842,739 confirmed COVID-19 cases had been reported around the world (WHO Coronavirus Dashboard, accessed: https://covid19.who.int) and 84,570 confirmed COVID-19 cases had been reported in China (National electronic government platform, accessed: http://gjzwfw.www.gov.cn/index.html). The distribution of the confirmed cases of COVID-19 in China and the population density of China are presented in Figure 1A. This raises health concerns for patients with multiple sclerosis (MS), an inflammatory demyelinating and neurodegenerative disorder of the central nervous system. Although MS primarily affects young adults, there are some patients older than 60 years with comorbidities such as hypertension and diabetes, a population with a demonstrated higher risk of severe disease and mortality from COVID-19 (1). MS-related functional limitations, such as inability to cough and clear the lungs, a condition associated with increased risk from infectious diseases (2), may increase the susceptibility to and severity of COVID-19. Furthermore, long-term use of disease-modifying therapies (DMTs) or immunosuppressants for MS may confer additional risk by suppressing the patient's immune status (3). Recently, several large cohort studies have been published regarding the association of DMTs and the severity of COVID-19 (4, 5). Anti-CD20 agent was reported significantly associated with increased risk of severe COVID-19 (6), while interferon β-1b (IFNB) (7) and teriflunomide (8–10) might benefit COVID-19 patients. On the other hand, viral infection itself and COVID-19 related treatment discontinuation could in turn increase the disease activity of MS (11). Therefore, balancing the risks of SARS-CoV-2 infection and MS disease activity in MS patients is a huge challenge for neurologists during the pandemic, and there is a significant need for reliable, real-world information about the impact of COVID-19 on MS patients.

Until now, few studies have explored COVID-19 susceptibility, severity, and mortality in MS patients, and most existing studies suggest that there is no association between DMT exposure and COVID-19 severity (3, 12, 13). However, there are limited data available to guide the management and clinical decision-making of MS patients during the COVID-19 pandemic. In addition, few studies focus on the impact of MS relapse brought on by COVID-19. To better understand the impact of COVID-19 on the MS population, we sent an online survey to all MS patients registered in our Multiple sclerosis and Neuromyelitis optica database (MSNMOBase) from 2011 to 2020. The goals of the survey and the current manuscript were to investigate both the morbidity of COVID-19 and the disease activity of MS in a cohort of MS patients during the pandemic.

## Materials and Methods

### Study Design and Population

This cross-sectional cohort study used data from the MSNMOBase, a hospital-based electronic database established in 2011 by the Center of Multiple Sclerosis and Related Disorders of Neurology Department in Peking Union Medical College Hospital (PUMCH). The patients registered in this database are from all over China (Figure 1B) and are followed annually or semiannually (14). One hundred and seventy-eight (49.3%) patients were on DMTs, including teriflunomide (*n* = 152), interferon β1-b (*n* = 18), and fingolimod (*n* = 8), while 56 (15.5%) patients were treated with immunosuppressants, including mycophenolate mofetil (*n* = 42), azathioprine (*n* = 11), cyclosporine (*n* = 1), cyclophosphamide (*n* = 1), and tacrolimus (*n* = 1) (Table 1).

**Table 1:** Demographic, clinical characteristics, and behavior of patients in the pandemic.

**Characteristics**	
No. of patients	361
Demographics	35.3 (28.75, 44.89)
Age during the pandemic, median (IQR), years	
Age ≥60 years old, No. (%)	12 (3.3)
Sex ratio, female: male	1.8:1
Disease duration, median (IQR), years	4.12 (2.13, 8.17)
EDSS score at last follow-up, median (IQR)	1.0 (0.0, 2.0)
Treatment during the pandemic, No. (%)	
Teriflunomide	152 (42.1)
Interferon beta	18 (5.0)
Fingolimod	8 (2.2)
Immunosuppressants^a^	56 (15.5)
None	127 (35.2)
Treatment duration, median (IQR), years	1.0 (0.49, 2.95)
Preventive approaches, No. (%)	
Avoiding nonessential outdoor activities	299 (82.8)
Using a mask	342 (94.7)
Washing hands frequently	307 (85.0)
Treatment disruption, No. (%)	49 (20.9)
Discontinued due to fear of hospital visits	28 (12.0)
Discontinued because of fear of infection	15 (6.4)
Reduced dose of DMTs because of failure to get medication	6 (2.6)

*DMTs, disease modifying therapies; EDSS, Expanded Disability Status Scale; IQR, interquartile range*.

a *Immunosuppressant: Mycophenolate mofetil (n = 42), Azathioprine (n = 11), Cyclosporine (n = 1), Cyclophosphamide (n = 1), Tacrolimus (n = 1)*.

On June 1, 2020, when there were no newly diagnosed COVID-19 cases in mainland China for 1 week, an online questionnaire was sent to all patients with MS in the MSNMOBase to investigate incidence and impact of the COVID-19 pandemic (from December 15, 2019, to June 1, 2020) in MS patients. The questionnaire mainly included the following four parts: the infection of COVID-19, preventive approaches used in the COVID-19 pandemic, major changes to medical care and MS treatments, and emotional response to the COVID-19 pandemic.

MS relapse was defined as new MS-related symptoms or worsening of existing symptoms of neurological dysfunction, lasting more than 24 h. The identification of relapses and dates of attacks during the COVID-19 pandemic were derived from medical records and confirmed by neurologists of the Center of Multiple Sclerosis and Related Disorders in PUMCH through a follow-up visit face-to-face visit in the clinic or an on-line video consultation.

**Figure 2:**
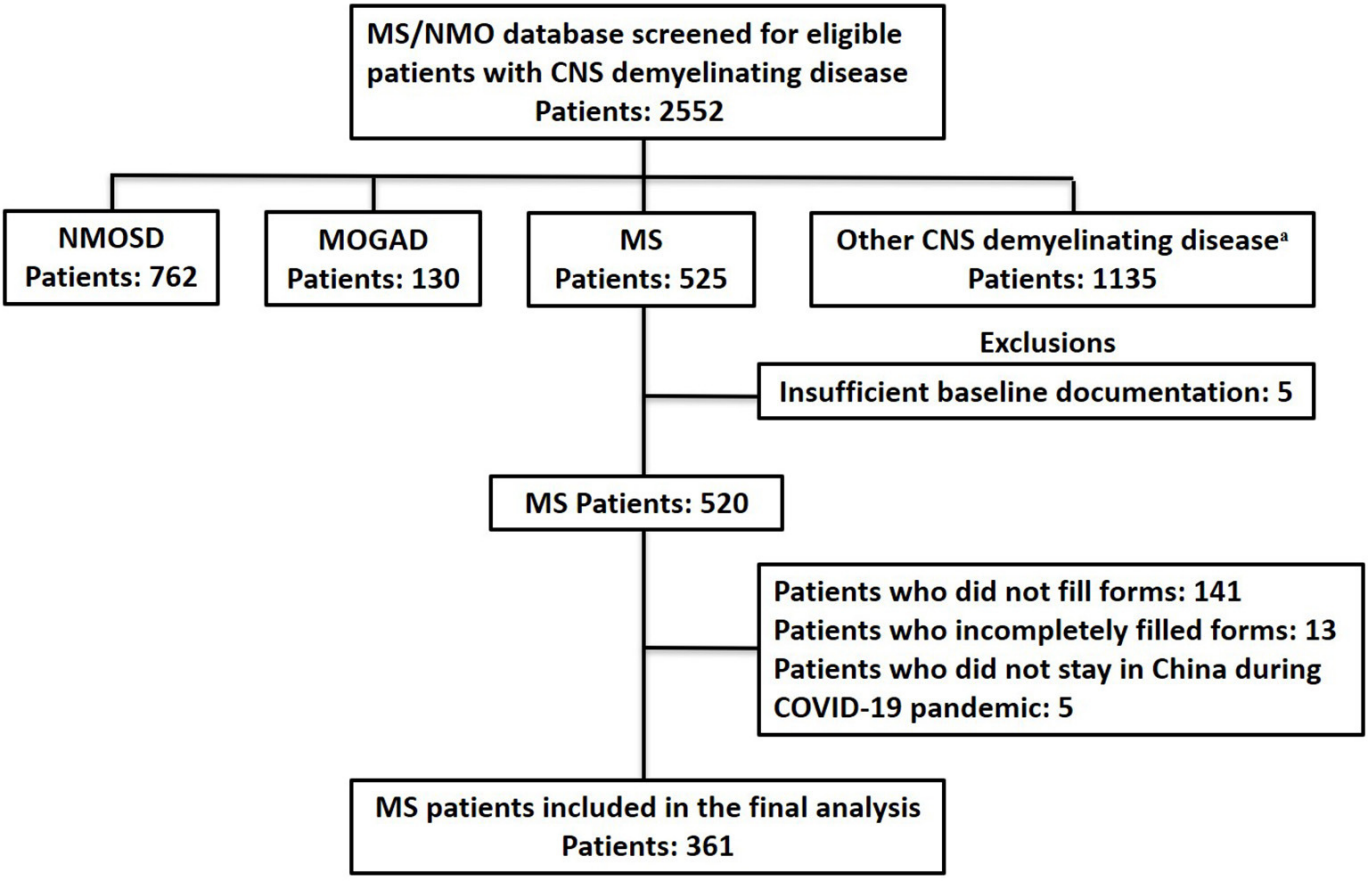
Flow chart of data processing and exclusions. COVID-19, coronavirus disease 2019; MOGAD, Myelin Oligodendrocyte Glycoprotein Antibody Associated Disorders; MS, Multiple Sclerosis; NMOSD, Neuromyelitis Optica Spectrum Disorder. ^a^Other CNS demyelinating disease: acute myelitis, brain stem encephalitis, aute disseminated encephalomyelitis, autoimmune GFAP astrocytopathy, and connective tissue disease.

Of 525 patients initially contacted, 384 responded to the questionnaires. After excluding patients who filled out the forms incompletely, had insufficient baseline documentation, or stayed abroad during the pandemic, 361 patients were included in the final statistical analysis; all of them were relapsing-onset MS and met the 2017 McDonald diagnostic criteria for MS (15) (Figure 2), including 329 relapsing remitting MS (RRMS) and 32 secondary progressive MS (SPMS). This study was approved by the ethics committee of PUMCH. All patients gave their written informed consent for the use of data for research purposes.

### Data Collection

Demographic data, dates of all MS attacks, MS attack-related clinical manifestations, Expanded Disability Status Scale (EDSS) scores, and treatments (drug names, dates of initiation, and termination) were collected during the patients' initial and regular clinical visits. Tests for complete blood count (CBC), lymphocyte subsets, and immunoglobin levels were performed in every regular visit. All of the above data were extracted from the MSNMOBase on June 15, 2020.

### Statistical Analysis

Descriptive statistics [percentage, median with interquartile range (IQR), or range and mean with standard deviation (SD)] were applied to describe results for baseline demographic data, baseline clinical characteristics, and patients' answers to the questionnaire. Student's *t*-test was used for the comparison of continuous variables, and a chi-square test or Wilcoxon test was used for the comparison of categorical data. Differences were considered significant when *P-*values were <0.05. All analyses were performed using SPSS software, version 26.0 (SPSS Inc., Chicago, IL, USA).

### Data Availability

The data that support the findings of this study are available from the corresponding author upon reasonable request.

## Results

### Baseline Patient Characteristics

Three hundred sixty-one patients with MS, including five from Hubei, were from 22 of 23 provinces, 4 of 5 autonomous regions, and 3 of 4 municipalities across China (Figure 1B). The demographic and clinical characteristics of the cohort are summarized in Table 1. Briefly, the median (IQR) age was 35.3 (28.8, 44.9) years, 12 (3.3%) were older than 60 years, 236 (65.4%) were women, and the median (IQR) disease duration was 4.1 (2.1, 8.2) years. The median (IQR) EDSS score at last follow-up was 1.0 (0.0, 2.0). Two hundred thirty-four (64.8%) patients received immunotherapy during the pandemic, including teriflunomide (*n* = 152), IFNB (*n* = 18), fingolimod (*n* = 8), and immunosuppressants (*n* = 56). The median (IQR) duration of treatment was 1.0 (0.5–3.0) years.

One hundred and fifty-nine patients with sufficient baseline documentation in the MSNMOBase were not included in the final analysis. We compared the baseline characteristics of these patients to the 361 patients included in final analysis and found no significant difference in age, gender, disease duration, EDSS score at last follow-up, immunotherapies, treatment duration, and ARR before treatment.

### Immune Status, Preventive Approaches, and COVID-19 Morbidity in MS Cohorts

**Figure 3:**
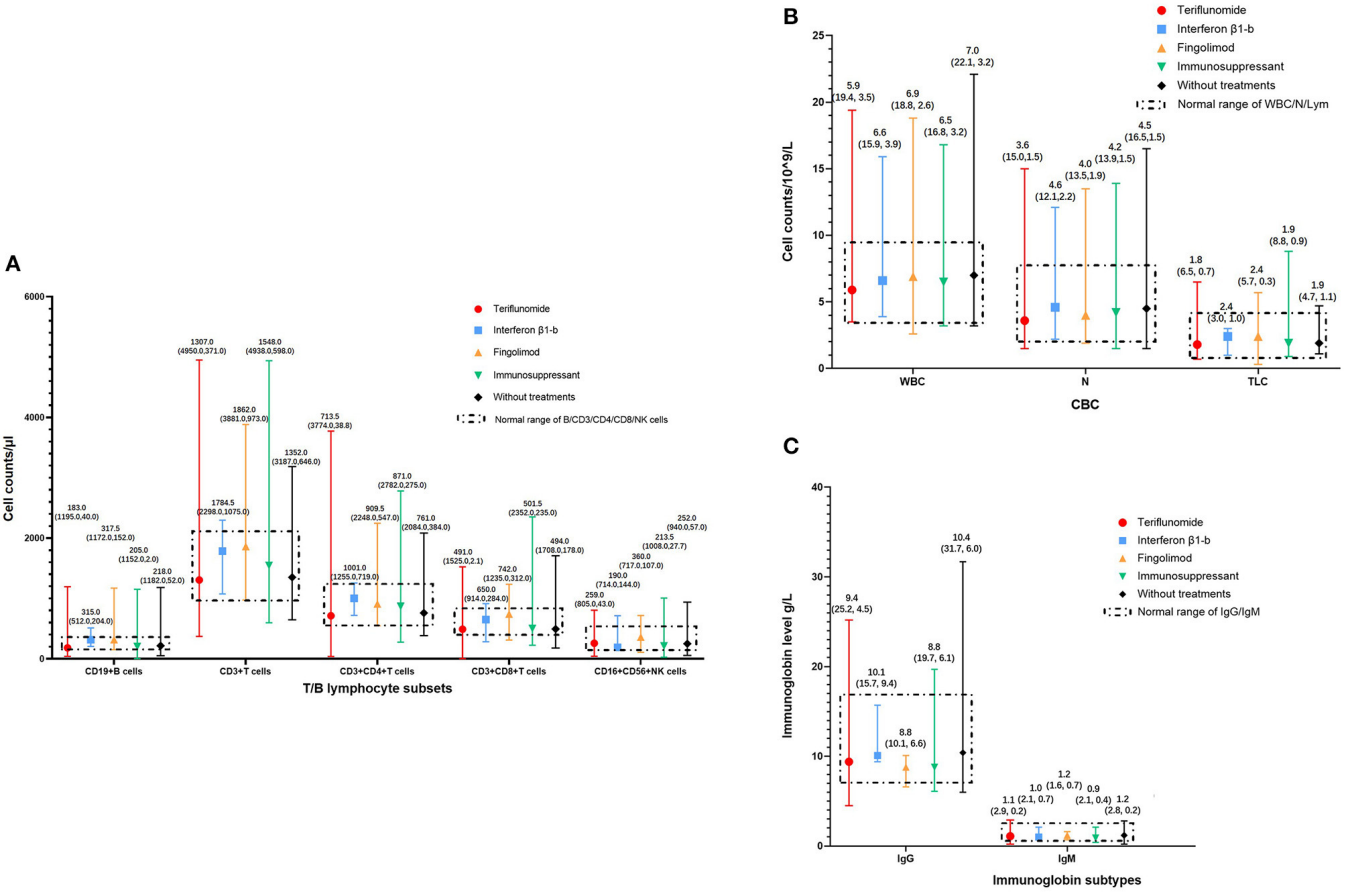
Immune status of MS patients on immunotherapies in COVID-19 pandemic. **(A)** T/B lymphocyte subsets of MS patients on immunotherapies in COVID-19 pandemic, categorized according to DMTs. **(B)** White blood cells, neutrophil granulocytes, and total lymphocyte counts of MS patients in COVID-19 pandemic, categorized according to DMTs. **(C)** Immunoglobin levels of MS patients in COVID-19 pandemic, categorized according to DMTs. CBC, Complete Blood Count; COVID-19, coronavirus disease 2019; DMTs, Disease-Modifying Therapies; Ig, Immunoglobin; MS, Multiple Sclerosis; N, neutrophil granulocytes; NK cells, Natural Killer cells; TLC, Total Lymphocyte Counts; WBC, White Blood Cells.

The complete blood cell (CBC) counts, lymphocyte subsets, and immunoglobulin levels within 6 months of the start of the pandemic were recorded in 272, 247, and 238 patients, respectively. Detailed median (range) levels of different immune cells and immunoglobulins in patients exposed to DMTs, patients exposed to immunosuppressants, and patients without immunotherapy are shown in Figure 3. Patients exposed to teriflunomide had the lowest median (range) levels of CD19^+^ B cells [183.0 (40.0–1,195.0)/μl], CD3^+^ T cells [1,307.0 (371.0–4,950.0)/μl], CD4^+^ T cells [713.5 (38.8–3,774.0)/μl], CD8^+^ T cells [491.0 (2.1–1,525.0)/μl], white blood cells [5.91 (3.51–19.36)/10^9^/L], neutrophil granulocytes [3.59 (1.49, 150.00)/10^9^/L], and total lymphocyte counts [1.82 (0.75–6.52)/10^9^/L]. Patients exposed to interferon β1-b had the lowest median (range) level of CD16^+^CD56^+^ NK cells [190.0 (144.0, 714.0)/μl] while patients exposed to immunosuppressants had the lowest median (range) level of immunoglobin G [8.75 (6.09, 19.66) g/L].

In patients exposed to DMTs, compared with interferon β1-b, patients exposed to teriflunomide had significantly lower median (range) levels of CD19^+^ B cells [315.0 (204.0, 512.0)/μl vs. 183.0 (40.0–1,195.0)/μl, *P* = 0.017] and CD4^+^ T cells [1,001.0 (719.0, 1,255.0)/μl vs. 713.5 (38.8–3,774.0)/μl, *P* = 0.026] while patients exposed to fingolimod had significantly lower median (range) levels of immunoglobin G [10.14 (9.44, 15.71) g/L vs. 8.80 (6.62, 10.09) g/L, *P* = 0.011]. The median (range) levels of CD3^+^ T cells, CD8^+^ T cells, CD16^+^ CD56^+^ NK cells, white blood cells, neutrophil granulocytes, and total lymphocyte counts had no significant difference among patients exposed to the three different DMTs. Patients exposed to DMTs had significantly lower median (range) levels of neutrophils than patients without immunotherapy [3.58 (1.49–13.46)/10^9^/L vs. 4.45 (1.52, 16.50)/10^9^/L, *P* < 0.01]. Compared with patients without immunotherapy, patients exposed to DMTs [9.43 (4.51–25.16) g/L vs. 10.39 (6.04–31.69) g/L, *P* < 0.01] and immunosuppressants [8.75 (6.09–19.66) g/L vs. 10.39 (6.04, 31.69) g/L, *P* < 0.05] had significantly lower median (range) levels of immunoglobulin G (Table 2).

**Table 2:** Immune status of patients with and without immunotherapy during the pandemic.

	**DMTs^a^**	**Immunosuppressant^b^**	**No treatment**	***P*-value**
**Levels of different immune cell counts**				
Neutrophile cell, median (range), 10^9^/L	3.58 (1.49, 13.46)	4.18 (1.52, 13.90)	4.45 (1.52, 16.50)	**0.008**
Lymphocyte, median (range), 10^9^/L	1.83 (0.29, 6.52)	1.86 (0.86,8.85)	1.91 (1.11, 4.68)	0.423
CD19^+^ B cell, median (range), ul	195.0 (40.0, 1,195.0)	205.0 (2.0, 1,152.0)	218.0 (52.0, 1,182.0)	0.239
CD3^+^CD4^+^ T cell, median (range), ul	718.0 (38.8, 3,774.0)	871.0 (275.0, 2,782.0)	761.0 (384.0, 2,084.0)	0.374
CD3^+^CD8^+^ T cell, median (range), ul	494.0 (2.1, 1,525.0)	501.5 (225.0, 2,352.0)	494.0 (178.0, 1,708.0)	0.607
**Levels of immunoglobulin G/M**				
Immunoglobulin G, median (range), g/L	9.43 (4.51, 25.16)	8.75 (6.09, 19.66)	10.39 (6.04, 31.69)	**0.004**
Immunoglobulin M, median (range), g/L	1.08 (0.23, 2.90)	0.93 (0.40, 2.09)	1.18 (0.17, 2.79)	0.352

*DMTs, disease modifying therapies*.

a *DMTs: Teriflunomide (n = 152), Interferon beta (n = 18), Fingolimod (n = 8)*.

b *Immunosuppressant: Mycophenolate mofetil (n = 42), Azathioprine (n = 11), Cyclosporine (n = 1), Cyclophosphamide (n = 1), Tacrolimus (n = 1)*.

The major COVID-19 preventive approaches used in our patient cohort included avoiding non-essential outdoor activities, using a mask, and washing hands frequently (Table 1). None of our MS patients were diagnosed with or suspected to have COVID-19, regardless of whether they received immunotherapies, were older than 60 years, or were from high-risk areas such as Hubei.

### The Impact of the COVID-19 Pandemic on Disease Activity

**Figure 4:**
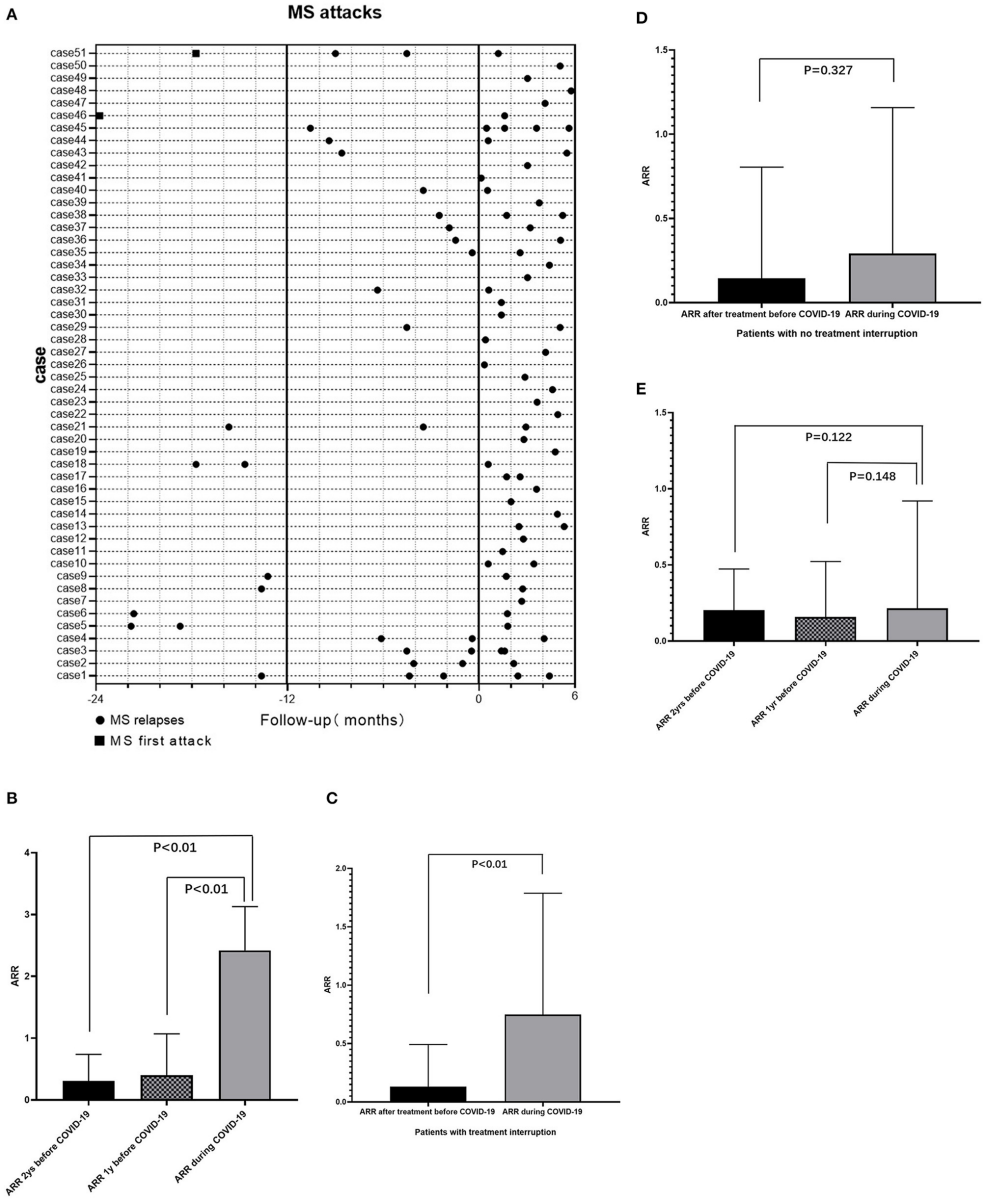
Impact of COVID-19 pandemic on MS attacks. **(A)** Relapses of 51 MS patients who had at least one relapse during pandemic and attacks of this group of patients in 1/2 years before COVID-19 pandemic. **(B)** For patients who had relapse during the pandemic (*n* = 51), compared with 1 year (*P* < 0.01) and 2 years (*P* < 0.01) before the pandemic, the ARR of patients during the pandemic increased significantly. **(C)** For patients with treatment interruption (*n* = 49), the ARR during the pandemic increased significantly compared with ARR after treatment but before the pandemic (*P* < 0.01). **(D)** For patients with continuous treatments during the pandemic (*n* = 185), compared to the ARR after treatment but before the pandemic, the ARR during the pandemic remained no significant change (*P* = 0.327). **(E)** For patients without immunotherapy (*n* = 127), there was no significant difference of ARR during the pandemic and 1 or 2 years before the pandemic. ARR, annualized relapse rate; COVID-19, coronavirus disease 2019; MS, Multiple Sclerosis.

Fifty-one (13.9%) patients had at least one relapse of MS during the COVID-19 pandemic between December 15, 2019, and June 1, 2020 (Figure 4A). Compared with 1 and 2 years before the pandemic, the annualized relapse rate (ARR) of these patients during the pandemic increased significantly, with a median (IQR) difference of 2.16 (1.16, 2.16) (*P* < 0.01) and 2.16 (1.66, 2.16) (*P* < 0.01), respectively (Figure 4B).

A total of 49 of 234 (20.9%) patients who were being treated with immunotherapy experienced treatment interruption during the pandemic. The ARR for these patients after treatment but before the pandemic was significantly lower than the ARR before treatment, with a median (IQR) difference of −0.947 (−3.071, −0.286) (*P* < 0.01). Forty (81.6%) of these patients had an ARR of 0 and nine (18.4%) patients had an ARR of 0–3 after treatment but before the pandemic, while only 32 (65.3%) of these patients had an ARR of 0 and up to 17 (34.7%) patients had an ARR of 0–3 during the pandemic. Hence ARR for these patients during the pandemic increased significantly compared to ARR after treatment but before the pandemic (*P* < 0.01) (Figure 4C). For 185 patients who experienced continuous immunotherapy during the pandemic, the ARR after treatment but before the pandemic decreased significantly compared to the ARR before treatment, with a median (IQR) difference of −0.714 (−1.605, −0.379) (*P* < 0.01). There was no significant change in ARR in this population during the pandemic (*P* = 0.327) (Figure 4D). For 127 patients without immunotherapy, there was no significant difference between ARR during the pandemic and ARR 1 or 2 years before the pandemic (Figure 4E).

Among patients with treatment interruption during the pandemic, 28 of 49 (12.0%) discontinued their treatment due to fear of hospital visits, 15 (6.4%) of them discontinued their treatments because of fear of SARS-CoV-2 infection, and 6 (2.6%) of them reduced their dose of DMTs because of failure to obtain medication.

## Discussion

This cross-sectional cohort study is, to our knowledge, the first observational study investigating both the morbidity of COVID-19 and relapses in a cohort of MS patients during the pandemic. We found that, with effective preventive approaches (reducing unnecessary outdoor activity, mask use, and frequent hand washing), the risk of SARS-CoV-2 infection did not increase in patients with MS, regardless of age or whether they had received immunotherapies during the pandemic. However, the disease activity of MS increased significantly due to treatment discontinuation caused by the COVID-19 pandemic.

The mechanisms of MS immunotherapies span a wide range of targets in the adaptive and innate immune systems, both of which are involved in the response to viral infections, including COVID-19 (16). Levels of specific lymphocyte subsets and immunoglobulins, such as CD3^+^CD4^+^ T cells, CD3^+^CD8^+^ T cells, and immunoglobulin G, are reported to be related to infection in chronic autoimmune diseases (17). As a result, it is hypothesized that MS patients receiving immunotherapy might have a higher risk of SARS-CoV-2 infection and unfavorable COVID-19 outcomes. Over 85% of our patients treated with DMTs were exposed to teriflunomide, which selectively and reversibly inhibits dihydro-orotate dehydrogenase and lymphocyte proliferation (18). Consistent with previous studies (19), we also found that patients exposed to DMTs in our cohort had significantly lower levels of neutrophils and immunoglobulin G. In addition, patients exposed to immunosuppressants had significantly lower levels of immunoglobulin G than patients who did not receive immunotherapy. However, even with this immunosuppressed status, none of our patients were diagnosed with or suspected of having COVID-19 under the current protective measures. This result is consistent with that of a previous multicenter survey that enrolled 1,804 MS patients in mainland China, none of whom contracted COVID-19 (12). Additionally, two previous studies on Caucasian patients with MS also showed that exposure to first-generation DMTs (such as IFNB and teriflunomide) was not associated with an increased risk of COVID-19 (20, 21). The relatively high level of adherence to the preventive protocols suggested by the World Health Organization (WHO)^2^ shown in our online survey has undoubtedly contributed to the absence of COVID-19 in our cohort.

Stressful events, such as natural disasters and certain life events, have been proven to induce immunological alterations (22–24), which led us to speculate that stress caused by the COVID-19 pandemic (25) could impact the course of MS. However, in this study, we observed a significantly increased relapse rate only in patients who experienced treatment disruption due to COVID-19, not in patients who received continuous treatment or who had no immunotherapy. These results emphasize the importance of ongoing treatment in MS patients during the pandemic. Similarly, Kanamori et al. (26) and Nisipeanu and Korczyn (27) reported no increased relapse in patients with MS exposed to the Great East Japan Earthquake in 2011 or the Persian Gulf War in 1991, respectively, and concluded that not all stress conditions increase exacerbations in patients with MS.

There is definitive evidence that the healthcare system was overwhelmed during the COVID-19 pandemic and that medical care for regular patients was negatively impacted (28, 29). Consequently, over 20% of our MS patients reported discontinuation or dose reduction of their DMTs due to failure to obtain medication during the pandemic and fear of infection or hospital visits, which might account for the high relapse rate during the COVID-19 pandemic. The global COVID-19 pandemic is expected to linger for possibly years, and neurologists must find the safest balance between protecting their patients from SARS-CoV-2 infection and ensuring the best care for patients with chronic diseases such as MS.

Our study has some limitations. First, although MS patients at our center were from all over China, this single-center study might not reflect the immune status of Chinese MS patients as a whole, and the number of patients from COVID-19 high-risk regions was not large. Second, we only analyzed patients who responded to the questionnaire. Although we did a descriptive analysis and found no significant differences of baseline characteristics between the patients included in the final analysis and ones who didn't, a selection bias might still exist. Third, since fingolimod was approved by the National Medical Products Administration (NMPA) not long before the pandemic, the number of patients exposed to fingolimod was rather small in our study, which might have limited the power to detect the impact of fingolimod on the immune status of MS patients. Fourth, lymphocytes depleting treatments, such as anti-CD20 therapies and alemtuzumab, which were believed to have a greater impact on infection severity, were not used in our MS patients. Last, the absence of cases of COVID-19 infection in our cohort could not lead to an estimation of the impact of DMTs on the risk or severity of infection. Besides, the observation period of our study is relatively short and the ARR regression toward the mean might not be completely excluded. Additional larger, multicenter, prospective cohort studies with a longer observational period are needed to further explore the impact of the COVID-19 pandemic on patients with MS.

## Conclusion

Despite some limitations, this observational cohort study suggests that continuing treatment with DMTs such as teriflunomide, IFNB, and fingolimod, as well as immunosuppressants such as mycophenolate mofetil and azathioprine, during the pandemic might be safe for patients with MS who use recommended COVID-19 preventive approaches, while withdrawal of the above treatments during the pandemic definitely increases MS disease activity.

## Data Availability Statement

The original contributions presented in the study are included in the article/supplementary material, further inquiries can be directed to the corresponding author/s.

## Ethics Statement

The studies involving human participants were reviewed and approved by The Institutional Review Board of Peking Union Medical College Hospital. All patients gave their written informed consent for participating in the present study and for the use of their data for publication.

## Author Contributions

YX, YZ, and HY contributed to the study concept and design. Data collection and analysis were performed by YZ, YX, TX, and HY. Statistical analysis was performed by YZ, YX, and TX. The first draft of the manuscript was written by YZ. Critical revisions of the manuscript for important intellectual content were performed by YX, BP, LC, and SZ. Study supervision was YX. All authors read and approved the final manuscript.

## Conflict of Interest

The authors declare that the research was conducted in the absence of any commercial or financial relationships that could be construed as a potential conflict of interest.

## Footnotes

^1^World Health Organization. (2020). Novel coronavirus situation report: 99. https://www.who.int/news-room/detail/28-04-2020-who-calls-for-healthy-safe-and-decent-working-conditions-for-all-health-workers-amidst-covid-19-pandemic [Accessed Apr 28, 2020].

^2^World Health Organization. (2020). Coronavirus disease 2019 (COVID-19) advice for the public. https://www.who.int/emergencies/diseases/novel-coronavirus-2019/advice-for-public [Accessed October 7, 2020].

## References

[1] Zhou F Yu T Du R Fan G Liu Y Liu Z. Clinical course and risk factors for mortality of adult inpatients with COVID-19 in Wuhan, China: a retrospective cohort study. Lancet. (2020) 395:1054–62. 10.1016/S0140-6736(20)30566-3 32171076 PMC7270627

[2] Montgomery S Hillert J Bahmanyar S. Hospital admission due to infections in multiple sclerosis patients. Eur J Neurol. (2013) 20:1153–60. 10.1111/ene.12130 23496086

[3] Bsteh G Bitschnau C Hegen H Auer M Pauli FD Rommer P. Multiple sclerosis and COVID-19: how many are at risk. Eur J Neurol. (2020). 10.1111/ene.14555. [Epub ahead of print]. 32978860 PMC7537184

[4] Salter A Fox RJ Newsome SD Halper J Li DKB Kanellis P. Outcomes and risk facters associated with SARS-CoV-2 infection in a North American Registry of patients with multiple sclerosis. JAMA Neurol. (2021). 10.1001/jamaneurol.2021.0688. [Epub ahead of print]. 33739362 PMC7980147

[5] Louapre C Collongues N Stankoff B Giannesini C Papeix C Bensa C. Clinical characteristics and outcomes in patients with coronavirus disease 2019 and multiple sclerosis. JAMA Neurol. (2020) 77:1079–88. 10.1001/jamaneurol.2020.2581 32589189 PMC7320356

[6] Sormani MP Rossi ND Schiavetti I Carmisciano L Cordioli C Moiola L. Disease-modifying therapies and coronavirus disease 2019 severity in multiple sclerosis. Ann Neurol. (2021) 89:780–9. 10.1002/ana.26028 33480077 PMC8013440

[7] Hung IF Lung KC Tso EY Liu R Chung TW Chu M. Triple combination of interferon beta-1b, lopinavir-ritonavir, and ribavirin in the treatment of patients admitted to hospital with COVID-19: an open-label, randomised, phase 2 trial. Lancet. (2020) 395:1695–704. 10.1016/S0140-6736(20)31042-4 32401715 PMC7211500

[8] Maghzi AH Houtchens MK Preziosa P Lonete C Beretich BD Stankiewicz JM. COVID-19 in teriflunomide-treated patients with multiple sclerosis. J Neurol. (2020) 267:2790–6. 10.1007/s00415-020-09944-8 32494856 PMC7268971

[9] Mantero V Baroncini D Balgera R Guaschino C Basilico P Annovazzi P. Mild COVID-19 infection in a group of teriflunomide-treated patients with multiple sclerosis. J Neurol. (2020). 10.1007/s00415-020-10196-9. [Epub ahead of print]. 32865629 PMC7457441

[10] Möhn N Saker F Bonda V Respondek G Bachmann M Stoll M. Mild COVID-19 symptoms despite treatment with teriflunomide and high-dose methylprednisolone due to multiple sclerosis relapse. J Neurol. (2020) 267:2803–5. 10.1007/s00415-020-09921-1 32494855 PMC7268187

[11] Sibley WA Bamford CR Clark K. Clinical viral infections and multiple sclerosis. Lancet. (1985) 1:1313–5. 10.1016/S0140-6736(85)92801-6 2860501 PMC7173199

[12] Fan M Qiu W Bu B Xu Y Yang H Huang D. Risk of COVID-19 infection in MS and neuromyelitis optica spectrum disease. Neurol Neuroimmunol Neuroinflamm. (2020) 7:e787. 10.1212/NXI.0000000000000787 32503092 PMC7286663

[13] Loonstra FC Hoitsma E van Kempen ZL Killestein J Mostert JP. COVID-19 in multiple sclerosis: the Dutch experience. Mult Scler. (2020) 26:1256–60. 10.1177/1352458520942198 32662742 PMC7493197

[14] Zhang Y Xu Y Xu T Yin H Zhu Y Peng B. Prediction of long-term disability in Chinese patients with multiple sclerosis: a prospective cohort study. Mult Scler Relat Disord. (2020) 46:102461. 10.1016/j.msard.2020.102461 32862039

[15] Thompson AJ Banwell BL Barkhof F Carroll WM Coetzee T Comi G. Diagnosis of multiple sclerosis: 2017 revisions of the McDonald criteria. Lancet Neurol. (2018) 17:162–73. 10.1016/S1474-4422(17)30470-2 29275977

[16] Shi Y Wang Y Shao C Huang J Gan J Huang X. COVID-19 infection: the perspectives on immune responses. Cell Death Differ. (2020) 27:1451–4. 10.1038/s41418-020-0530-3 32205856 PMC7091918

[17] Wu L Wang X Chen F Lv X Sun W Guo Y. T cell subsets and immunoglobulin G levels are associated with the infection status of systemic lupus erythematosus patients. Braz J Med Biol Res. (2017) 51:e4547. 10.1590/1414-431x20154547 29267496 PMC5731325

[18] Bar-Or A Pachner A Menguy-Vacheron F Kaplan J Wiendl H. Teriflunomide and its mechanism of action in multiple sclerosis. Drugs. (2014) 74:659–74. 10.1007/s40265-014-0212-x 24740824 PMC4003395

[19] O'Connor P Comi G Freedman MS Miller AE Kappos L Bouchard J. Long-term safety and efficacy of teriflunomide: nine-year follow-up of the randomized TEMSO study. Neurology. (2016) 86:920–30. 10.1212/WNL.0000000000002441 26865517 PMC4782117

[20] Amor S Baker D Khoury SJ Schmierer K Giovanonni G. SARS-CoV-2 and multiple sclerosis: not all immune depleting DMTs are equal or bad. Ann Neurol. (2020) 87:794–7. 10.1002/ana.25770 32383812 PMC7273059

[21] Mansoor S Kelly S Murphy K Waters A Siddiqui NS. COVID-19 pandemic and the risk of infection in multiple sclerosis patients on disease modifying therapies: “what the bleep do we know?”. Egypt J Neurol Psychiatr Neurosurg. (2020) 56:44. 10.1186/s41983-020-00177-0 32372857 PMC7194245

[22] Abdollahpour I Nedjat S Mansournia MA Eckert S Weinstock-Guttman B. Stress-full life events and multiple sclerosis: a population-based incident case-control study. Mult Scler Relat Disord. (2018) 26:168–72. 10.1016/j.msard.2018.09.026 30268037

[23] AlZahrani AS Alshamrani FJ Al-Khamis FA Al-Sulaiman AA Ghamdi WS Ghamdi OA. Association of acute stress with multiple sclerosis onset and relapse in Saudi Arabia. Saudi Med J. (2019) 40:372–8. 10.15537/smj.2019.4.24010 30957131 PMC6506663

[24] Brown RF Tennant CC Dunn SM Pollard JD. A review of stress-relapse interactions in multiple sclerosis: important features and stress-mediating and -moderating variables. Mult Scler. (2005) 11:477–84. 10.1191/1352458505ms1170oa 16042233

[25] Cooke JE Eirich R Racine N Madigan S. Prevalence of posttraumatic and general psychological stress during COVID-19: a rapid review and meta-analysis. Psychiatry Res. (2020) 292:113347. 10.1016/j.psychres.2020.113347 32763477 PMC7392847

[26] Kanamori Y Nakashima I Takai Y Misu T Kuroda H Nishiyama S. Impact of the great east Japan earthquake in 2011 on MS and NMOSD: a study in Sendai, Japan. J Neurol Neurosurg Psychiatry. (2017) 88:362–4. 10.1136/jnnp-2016-313890 27535942

[27] Nisipeanu P Korczyn AD. Psychological stress as risk factor for exacerbations in multiple sclerosis. Neurology. (1993) 43:1311–2. 10.1212/WNL.43.7.1311 8327130

[28] Horisberger A Moi L Ribi C Comte D. Impact of COVID-19 pandemic on SLE: beyond the risk of infection. Lupus Sci Med. (2020) 7:e000408. 10.1136/lupus-2020-000408 32376774 PMC7223264

[29] Zhao J Li H Kung D Fisher M Shen Ying. Impact of the COVID-19 epidemic on stroke care and potential solutions. Stroke. (2020) 51:1996–2001. 10.1161/STROKEAHA.120.030225 32432997 PMC7258753

